# Supplementary material for: Causal Relationship between Aging and Anorexia Nervosa: A White-Matter-Microstructure-Mediated Mendelian Randomization Analysis
Source: Biomedicines. 2024 Aug 16;12(8):1874. doi: 10.3390/biomedicines12081874 (PMC11351342; doi:10.3390/biomedicines12081874)
Supplement: Supplementary file 1 [file biomedicines-12-01874-s001.zip › Supplementary Figure/Figure S8/Leaveoneout plot of MR analysis for TL to ubm-b-2003.pdf]

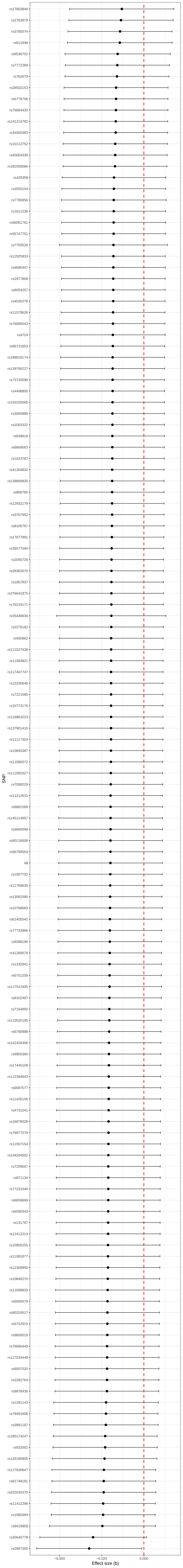

rs17803849
rs2763979
rs3785074
rs611646
rs6536702
rs7772289
rs762679
rs28502153
rs6776756
rs75664430
rs141214782
rs34550383
rs10112752
rs45604339
rs182059586
rs429358
rs2555104
rs7790856
rs1611236
rs56061761
rs55747751
rs7705526
rs12925933
rs4695407
rs2977608
rs6054257
rs4530278
rs11579626
rs76065543
rs4724
rs66731853
rs188918174
rs139795227
rs73730598
rs4498805
rs150150565
rs3093888
rs1003322
rs939916
rs6669563
rs1023767
rs41304832
rs139669835
rs869785
rs12932179
rs3767952
rs8105767
rs17677991
rs28577594
rs2056726
rs28363070
rs1957937
rs376641875
rs76219171
rs35446936
rs2276182
rs450962
rs111527438
rs11584821
rs117407747
rs13230646
rs7221585
rs10773176
rs116863223
rs137901416
rs11117354
rs10845387
rs11085072
rs111950327
rs7099229
rs11212631
rs6881568
rs145114957
rs9940099
rs80116508
rs56799554
All
rs1907702
rs11769630
rs13062095
rs10768683
rs61405042
rs77732866
rs9398196
rs41269079
rs1332941
rs6751209
rs117512405
rs8102497
rs7164950
rs113525195
rs6790988
rs142426306
rs9955360
rs17445108
rs112394943
rs6587577
rs11426156
rs4731541
rs16978028
rs79977579
rs11557154
rs144204502
rs7209057
rs871134
rs77231040
rs6659669
rs6590343
rs131797
rs12412214
rs10905255
rs11991877
rs12369950
rs10840270
rs11699829
rs6584579
rs80324517
rs5742915
rs9600019
rs76666449
rs117034449
rs6007020
rs2282764
rs9878436
rs1291143
rs78491606
rs3891167
rs185174247
rs932002
rs143190905
rs117630647
rs61748181
rs202034370
rs11412296
rs1985369
rs9419958
rs35640778
rs2967355
SNP
−0.050
−0.025
0.000
Effect size (b)
